# Synthesis and characterization of emamectin-benzoate slow-release microspheres with different surfactants

**DOI:** 10.1038/s41598-017-12724-6

**Published:** 2017-10-06

**Authors:** Yan Wang, Anqi Wang, Chunxin Wang, Bo Cui, Changjiao Sun, Xiang Zhao, Zhanghua Zeng, Yue Shen, Fei Gao, Guoqiang Liu, Haixin Cui

**Affiliations:** 1 Institute of Environment and Sustainable Development in Agriculture, Chinese Academic of Agriculture Sciences, Beijing, 100081 China; 2 Nano Agricultural Research Center, Chinese Academic of Agriculture Sciences, Beijing, 100081 China

## Abstract

Pesticide slow-release formulations provide a way to increase the efficiency of active components by reducing the amount of pesticide that needs to be applied. Slow-release formulations also increase the stability and prolong the control effect of photosensitive pesticides. Surfactants are an indispensable part of pesticide formulations, and the choice of surfactant can strongly affect formulation performance. In this study, emamectin-benzoate (EMB) slow-release microspheres were prepared by the microemulsion polymerization method. We explored the effect of different surfactants on the particle size and dispersity of EMB in slow-release microspheres. The results indicated that the samples had uniform spherical shapes with an average diameter of 320.5 ±5.24 nm and good dispersity in the optimal formulation with the polymeric stabilizer polyvinyl alcohol (PVA) and composite non-ionic surfactant polyoxyethylene castor oil (EL-﻿40). The optimal EMB pesticide slow-release microspheres had excellent anti-photolysis performance, stability, controlled release properties, and good leaf distribution. These results demonstrated that EMB slow-release microspheres are an attractive candidate for improving pesticide efficacy and prolonging the control effect of EMB in the environment.

## Introduction

Pesticides play an important role in agriculture by preventing crop loss caused by major diseases and pests. The effective availabilities of traditional pesticide formulations are usually less than 30% due to losses caused by poor dispersion, droplet drift, and biodegradation, among other factors. As a result, the ultimate rate of pesticides reaching target pests is often below 0.1%^1,2^. The loss of large amounts of pesticides in non-target areas is a serious threat to food security and the environment^3,4^. Therefore, it is important for researchers to improve the usage rate of pesticides and to extend their duration of activity in the environment^5–10^.

Synthesis and development of advanced pesticide formulations has become necessary to increase the effectiveness of pesticides in the field^11–13^. Despite the fact that a sufficient amount of pesticides are typically applied during spraying, pesticide microemulsions (ME), water dispersible granules (WDG), and other traditional pesticide formulations are usually below the effective concentration for controlling pests within a short period of time^1,5,7,8^. This problem occurs due to the loss and degradation of active ingredients from photolysis, hydrolysis, microbiological degradation, or oxidation/reduction. Consequently, only a small amount of pesticide that is applied produces the desired control effect. The construction of pesticide slow-release formulations could effectively reduce volatilization, degradation, and loss of pesticides to non-target environments, and allow for sustained and effective release of active ingredients over a longer period of time^14–16^. Pesticide slow-release formulations are currently one of the most effective approaches to improving pesticide usage rates^17–23^.

Recently, the development of nanomaterials and related technologies has provided new ideas for the creation of pesticide slow-release formulations^24–28^. Using nanomaterials to encapsulate pesticides through absorption, coupling, and packaging could allow for pesticide slow-release formulations with different structures^29^. In particular, effective and broad-spectrum biogenic pesticides, such as emamectin benzoate (EMB; (4″R)-4″-deoxy-4″-(methylamino)-avermectin B1 benzoate, chemical structure shown in Supplementary Fig. 1, have high insecticidal activity, and can target pests with ultra-high efficiency and with low toxicity to mammals and human beings^30–34^. However, some characteristics of EMB, including short persistent effect, photolysis, and soil absorption have caused problems during application. Encapsulation of EMB into specific carriers to create a slow-release system can prevent direct exposure of active ingredients to the environment, reducing evaporation and degradation of EMB^30–34^. In addition, the particle size of the slow-release system also influences pesticide activity and usage rage^5^. Pesticides with a small particle size have the benefit of good distribution and sufficient contact with crop leaf surfaces, which means that small particles can enhance deposition and permeation on leaf surfaces, thus enhancing the pesticide effect and usage rate^7–9^. Surfactants are one of the main factors affecting size during synthesis of pesticide slow-release particles^8,9^. However, there are few reports on the influence of different surfactants on pesticide slow-release formulations.

To study the effect of surfactants on slow-release formulations of pesticides, we constructed EMB slow-release microspheres using the biodegradable polymer, polylactic acid (PLA), as the carrier. The influence of ten different varieties of surfactants on the size of EMB microspheres was investigated and the optimized formulation was screened. We also investigated the morphology and structure, slow-release property, stability, anti-photolysis capability, and foliar deposition of the EMB microsphere. The results showed that the optimal EMB slow-release microsphere effectively improved the photostability of the active pesticide ingredient. In addition, the EMB microsphere had a clear slow-release property and good surface deposition, both of which are important for increasing the duration of EMB in the environment and improving the pesticide usage rate.

## Results and Discussion

### Preparation of the EMB slow-release microsphere

**Figure 1:**
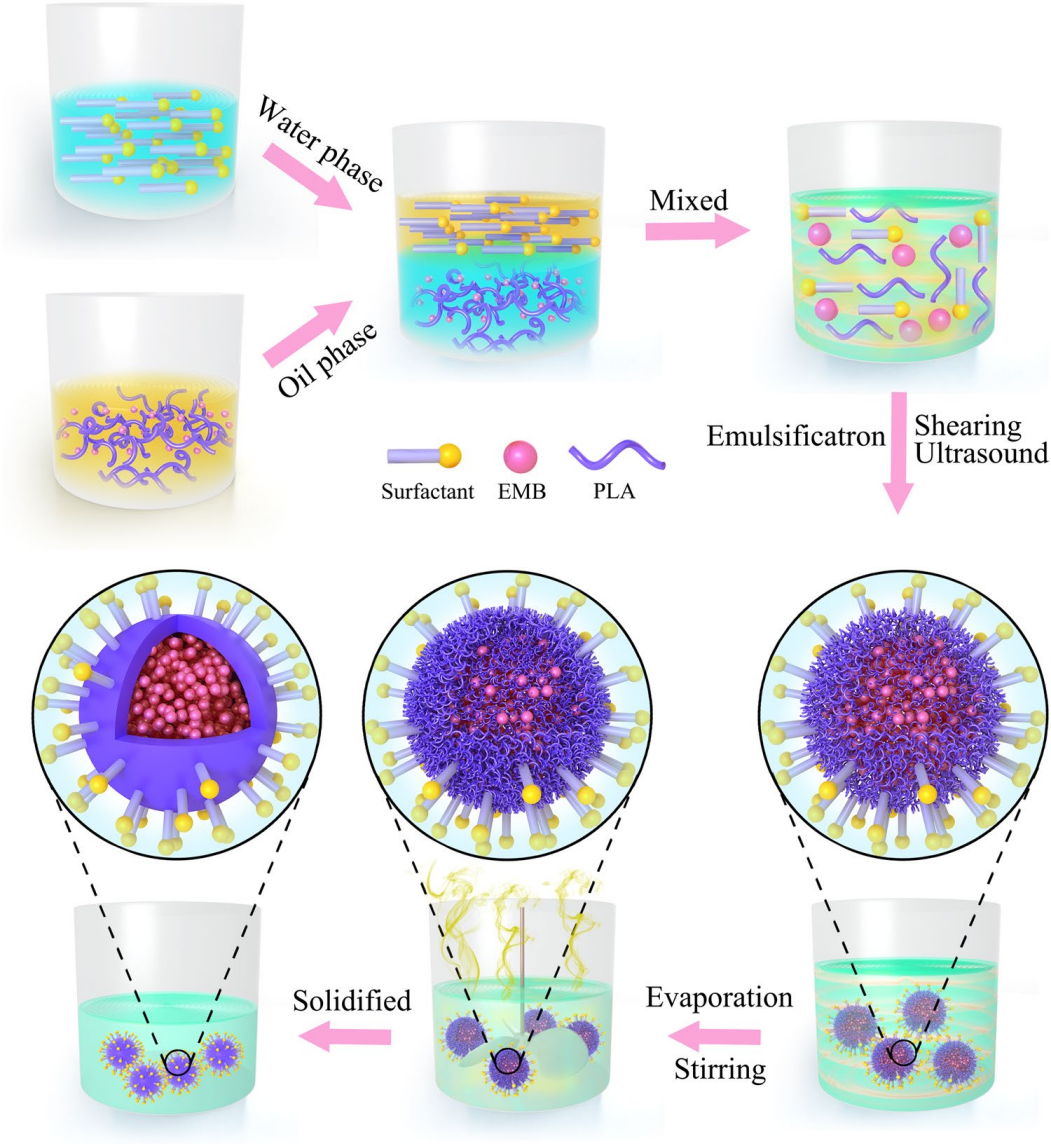
Schematic description of preparing the EMB slow-release microspheres.

EMB is a broad-spectrum, highly effective pesticide but its sensitivity to environmental conditions and easy adsorption in soil have limited its application. At present, the traditional commercial EMB formulations mainly include emulsifiable concentrate (EC), suspension concentrate (SC), and microemulsion (ME). These traditional EMB formulations are open systems that result in a short duration in the field and low utilization efficiency. Here, we used the safe, environmentally-friendly, and biodegradable polymer material PLA as a pesticide carrier to load EMB, and prepared EMB slow-release microspheres using an emulsion polymerization method combined with physical dispersion technology (Fig. 1). To ensure effective encapsulation, pesticides should be dispersed into small droplets so they are stable in the dispersion phase, prior to being effectively packaged in carrier materials. Surfactants have an important effect on the morphology, average size, dispersity, and stability of the pesticide system. Poly(vinyl alcohol) (PVA) is a polyhydroxy compound that is commonly used as an emulsifier with surface activity, emulsifying ability, and dispersing ability. PVA also has a thickening effect as a colloid-protecting agent. Aqueous solutions of PVA are widely used as stabilizers in emulsion polymerization, and they can improve the stability of emulsion polymerization systems. However, PVA contains too many hydrophilic groups and surface activity is relatively low. Combining PVA with other surfactants can effectively improve surface activity. Therefore, in order to obtain the optimal particle size and dispersibility, we chose PVA as the stabilizer and investigated the cooperative effects of PVA with other surfactants in our experiment.

### The influence of surfactant on particle size and dispersibility

**Figure 2:**
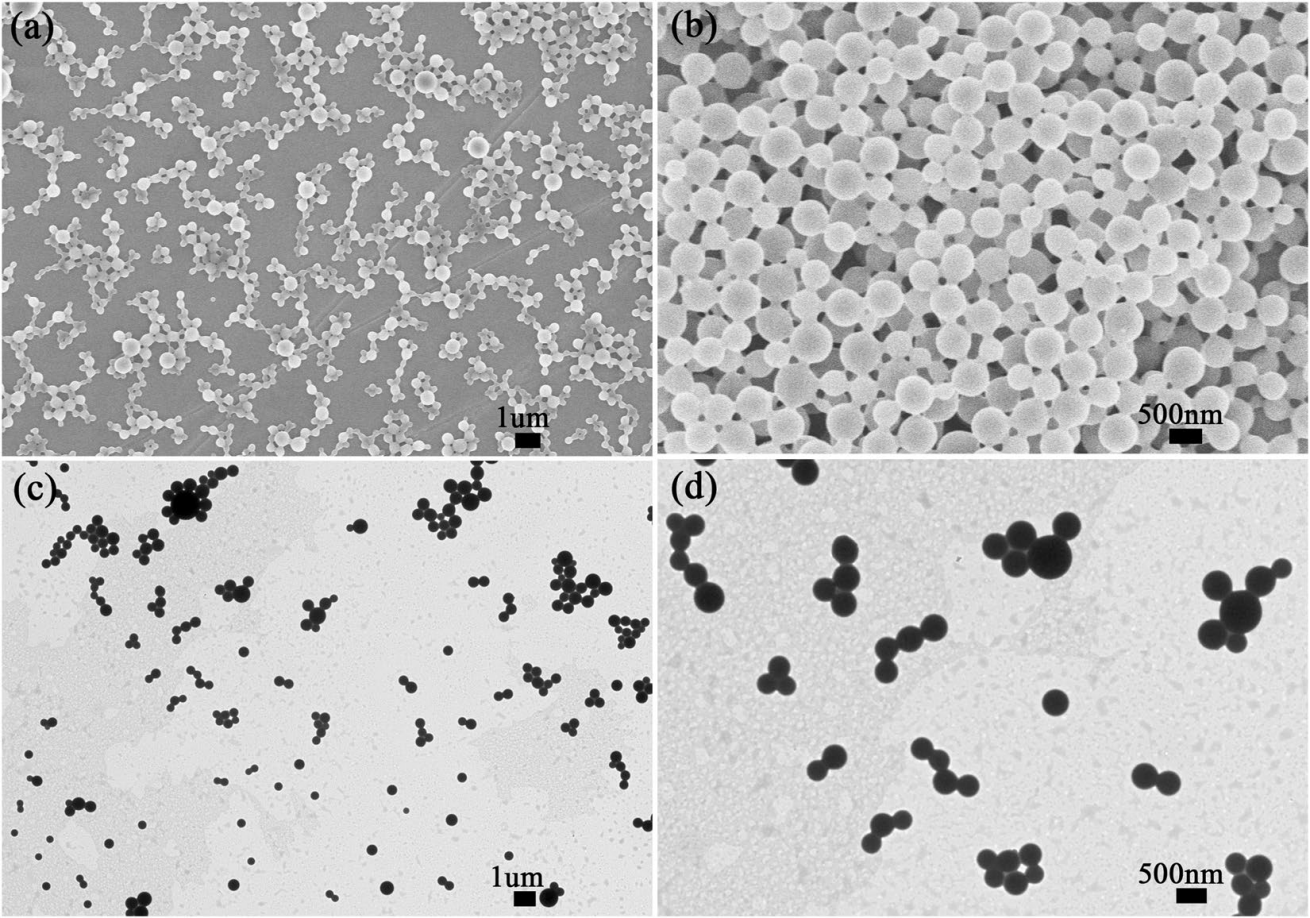
SEM images (**a**,**b**) and TEM images (**c**,**d**) of the EMB slow-release microspheres stabilized by EL-40 at different magnifications.

**Figure 3:**
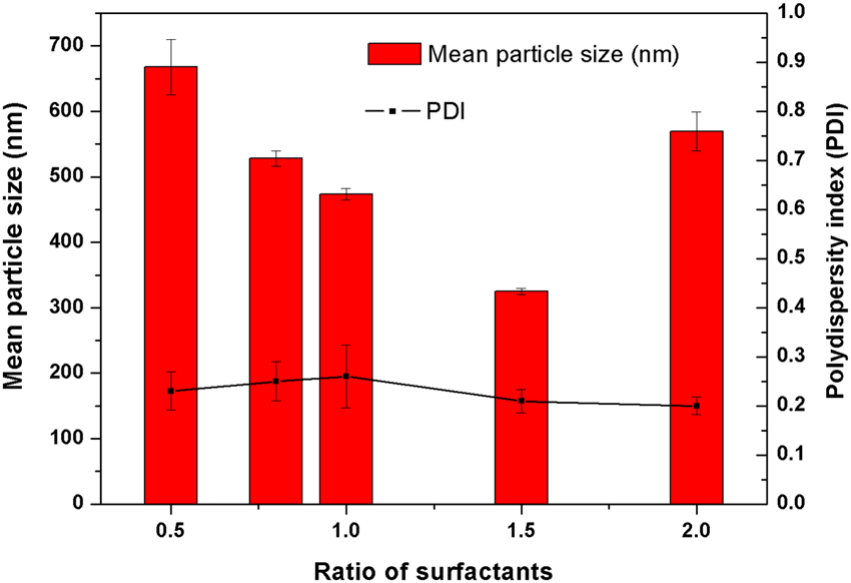
The influence of mass ratio of PVA/EL-40 on the mean particle size and PDI of the EMB slow-release microsphere.

**Figure 4:**
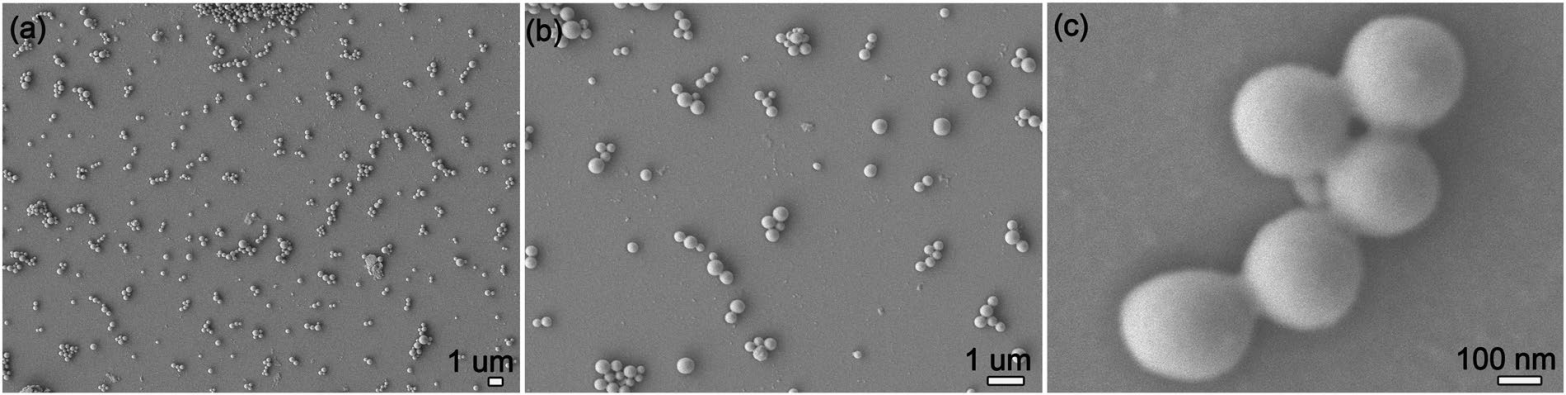
SEM images of the optimal EMB slow-release microsphere with the mass ratio 1.5 of PVA/EL-40 at different magnifications.

We investigated the influence of surfactant on the particle size and dispersibility of the EMB slow-release microsphere. Table 1 shows the average particle sizes and polydispersity index (PDI) of the EMB slow-release microsphere stabilized with different surfactants. The results showed that the EMB slow-release microsphere prepared with EL-40 is optimal; average particle sizes of the EL formulation were less than 500 nm. The PDI value of samples prepared with EL-40 was 0.259 ± 0.064, which reflects good uniformity and stability for the system^35,36^. EL-40 has a better dispersion effect as a typical nonionic surfactant in emulsion system than anionic surfactants. The anionic surfactant may induce particle aggregation via electrostatic interaction due to positively charge of EMB in solution^9^. EL-40 has a more appropriate hydrophile-lipophile balance (HLB) value of 13–14 for the o/w emulsion system, and therefore the EL-stabilized microspheres have relatively small particle size. The SEM (Fig. 2a,b) and TEM (Fig. 2c,d) images showed that the EMB slow-release microsphere with EL-40 as a surfactant had an average diameter of 473.47 ± 8.49 nm with uniform spherical shapes, and a smooth surface. Small pesticide particles have the benefits of good permeability, adhesion, and spreading properties, and thus enhance the pesticide effect and usage rate. In order to further optimize the EMB microsphere, we also investigated the effect of the mass ratio of PVA/EL-40 on the particle size and on PDI (Fig. 3). Results showed that the microsphere size could be further decreased to 320.5±5.24 nm with a PVA/EL-40 mass ratio of 1.5, and PDI less than 0.3, indicating a good size distribution. Supplementary Fig. 2 and Fig. 4 show the TEM image and size distribution of the EL-stabilized microsphere with the optimal PVA/EL-40 mass ratio, which illustrates that the EMB microsphere has good dispersity and size distribution under different electron microscope magnification levels. The loading content and entrapment efficiency of EMB in the EL-stabilized microsphere were also measured. The results indicated that the specimen had loading content of 40.8% and entrapment efficiency of higher than 83.2%. High pesticide loading content and entrapment efficiency in carriers are extremely desirable for the design of efficient pesticide formulations. It would not only save time, manpower, and resources during the preparation process but also avoid extensive use in the spraying process and reduce environmental pollution^5^.

**Table 1:** The influence of surfactant on the average particle size and PDI of the EMB slow-release microsphere.

Surfactant	Mean size (nm) ± S.D.	PDI ± S.D.
EL 40	473.47 ± 8.49	0.259 ± 0.064
Span 60	3227.00 ± 440.94	0.359 ± 0.111
Tween 80	649.80 ± 9.81	0.221 ± 0.007
Span 80	1271.33 ± 53.53	1.000 ± 0.000
Tween 60	623.33 ± 20.37	0.270 ± 0.090
Emulsifier 500	752.60 ± 43.69	0.202 ± 0.060
Emulsifier 600	519.37 ± 23.27	0.238 ± 0.088
Emulsifier 700	596.30 ± 14.96	0.227 ± 0.039
Emulsifier 1601	1116.37 ± 138.45	0.250 ± 0.031
AEO-9	1537.33 ± 93.66	0.203 ± 0.102

S.D: standard deviation of three measurements.

### Infrared spectrum analysis

**Figure 5:**
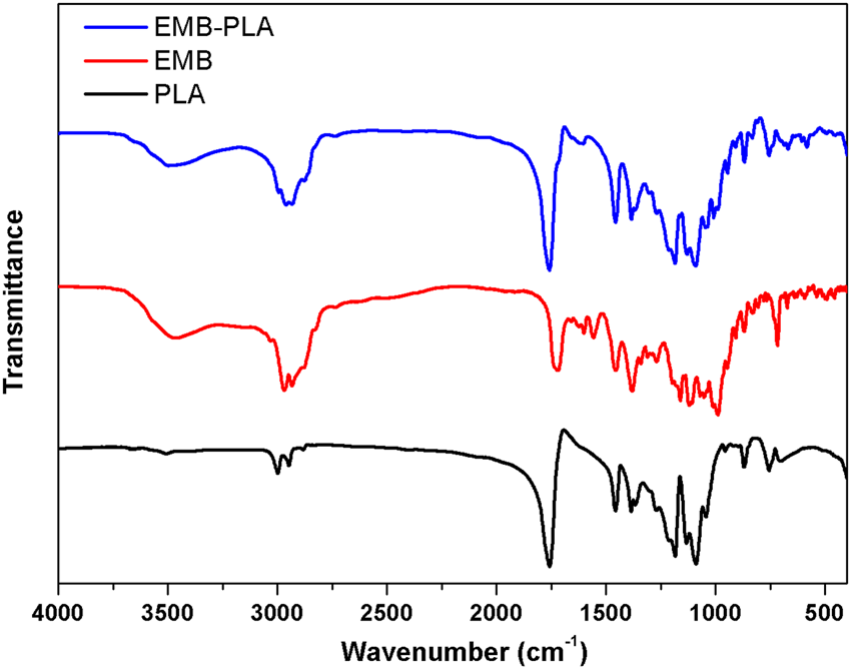
FT-IR spectra of PLA, EMB, and the optimal EMB slow-release microsphere (EMB-PLA).

Fourier transform-infrared (FT-IR) measurement was used to verify that EMB was encapsulated in the slow-release microsphere. Fig. 5 shows the FT-IR spectra of the technical EMB, blank PLA, and the EMB-PLA slow-release microsphere. The typical absorption peaks at 1731 cm^−1^ for C=O, 3459 cm^−1^ for OH, and 1608 cm^−1^ for benzene skeleton vibration appear in the spectra of EMB. For PLA, 1758 cm^−1^ is attributed to the C=O stretching vibration peak and 1086 cm^−1^ is attributed to the COC stretching vibration peak. Microspheres showed strong peaks at 3459 cm^−1^, 1608 cm^−1^, 1086 cm^−1^, as well as 1758 cm^−1^, which were characteristic bands of EMB and PLA (Fig. 5). The results indicated that EMB was encapsulated successfully into the PLA carriers to form the EMB slow-release microsphere.

### Slow release property of the EMB microsphere

**Figure 6:**
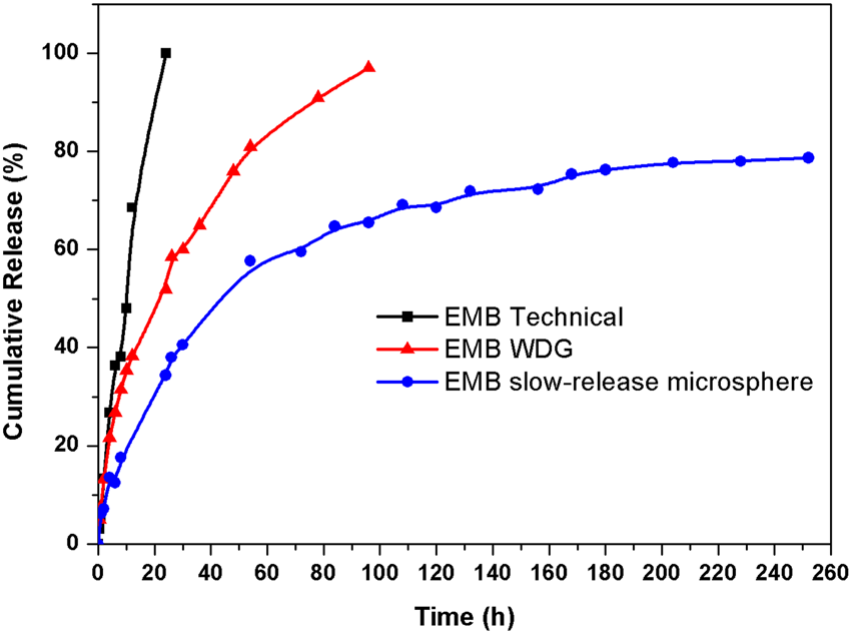
Cumulative release profiles of the optimal EMB slow-release microsphere using the commercial EMB WDG and technical EMB as controls .

Developing a pesticide formulation with controlled and slow release properties is essential for environmentally sensitive active ingredients because of their short active duration. The release behaviours of EMB slow-release microspheres were investigated using the technical EMB and commercial WDG as controls (Fig. 6). EMB technical was completely released after 24 h, and the cumulative release of commercial WDG reached 97% after 96 h. Compared with EMB technical and commercial WDG, the microsphere released EMB at relatively slow speeds and maintained its sustained release for longer periods. The EMB release profiles from the microsphere consisted of a relatively short burst release followed by a gradual release phase over 200 hours. These properties could not only increase the efficiency of pesticide applications and decrease the spraying dosage but also reduce environmental pollution.

### Stability evaluation

**Figure 7:**
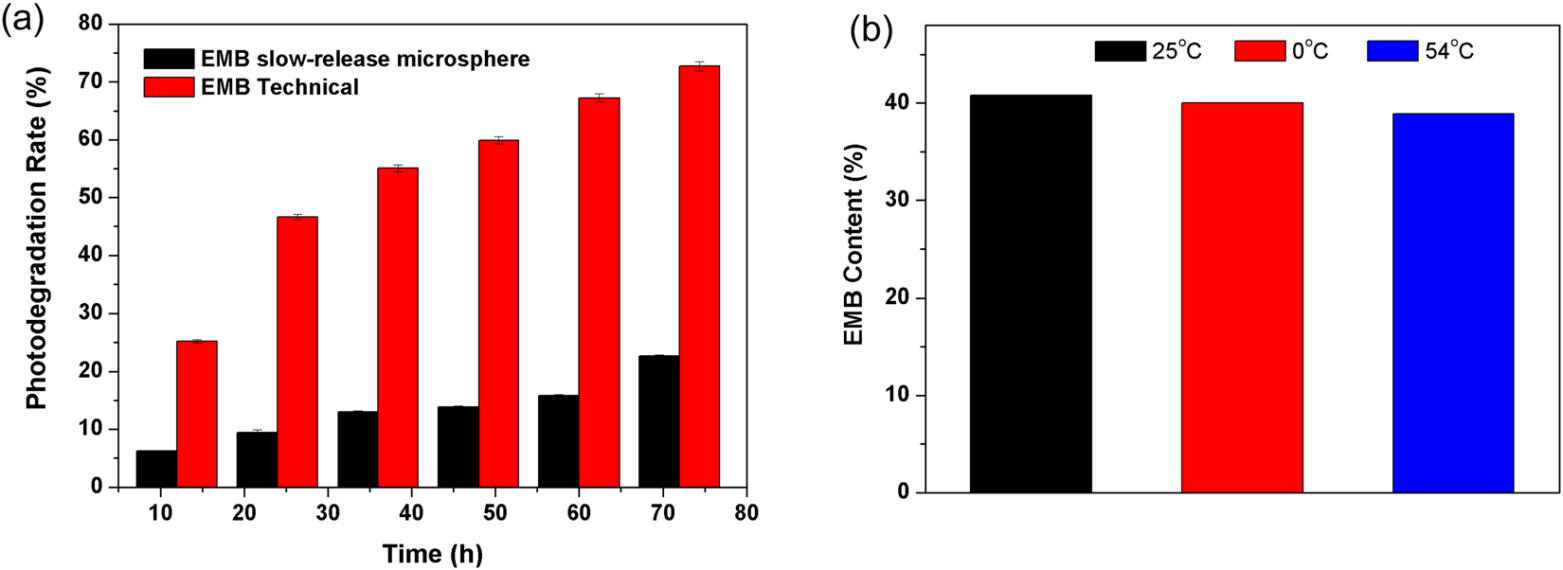
Comparison of the EMB photolysis percentage of the EMB microsphere and the technical EMB under UV irradiation (**a**), and EMB contents of the EMB slow-release microsphere before and after 0 °C for 7 days and 54 °C for 14 days (**b**).

To verify the anti-photolysis performance of EMB in the slow-release microsphere, the photolytic rate of EMB was estimated by artificial irradiation. Analysis of the photolysis rate of EMB to irradiation time is shown in Fig. 7a. The photolytic percentages of EMB were 13.06% and 55.05% for the microsphere and the EMB technical after 36 h, respectively, and were 22.65% and 72.74%, respectively, after 72 h. The results of UV irradiation confirmed that the microsphere could significantly prevent photolysis of EMB, resulting from the protective effect of the carrier wall. The storage stability of the microsphere was also evaluated by measuring EMB content at different temperatures (25 °C, 0 °C, 54 °C; Fig. 7b). Results showed that the microsphere remained stable with no major changes in the loading content during storage at room temperature or 0 °C. A small loss of EMB was observed after 14 days at 54 °C due to the accelerated degradation of EMB at higher temperatures (Fig. 7b). These results show that the EMB slow-release microsphere can be kept in a relatively stable state during storage.

### Dispersion behaviour and contact angle on cucumber leaf

**Figure 8:**
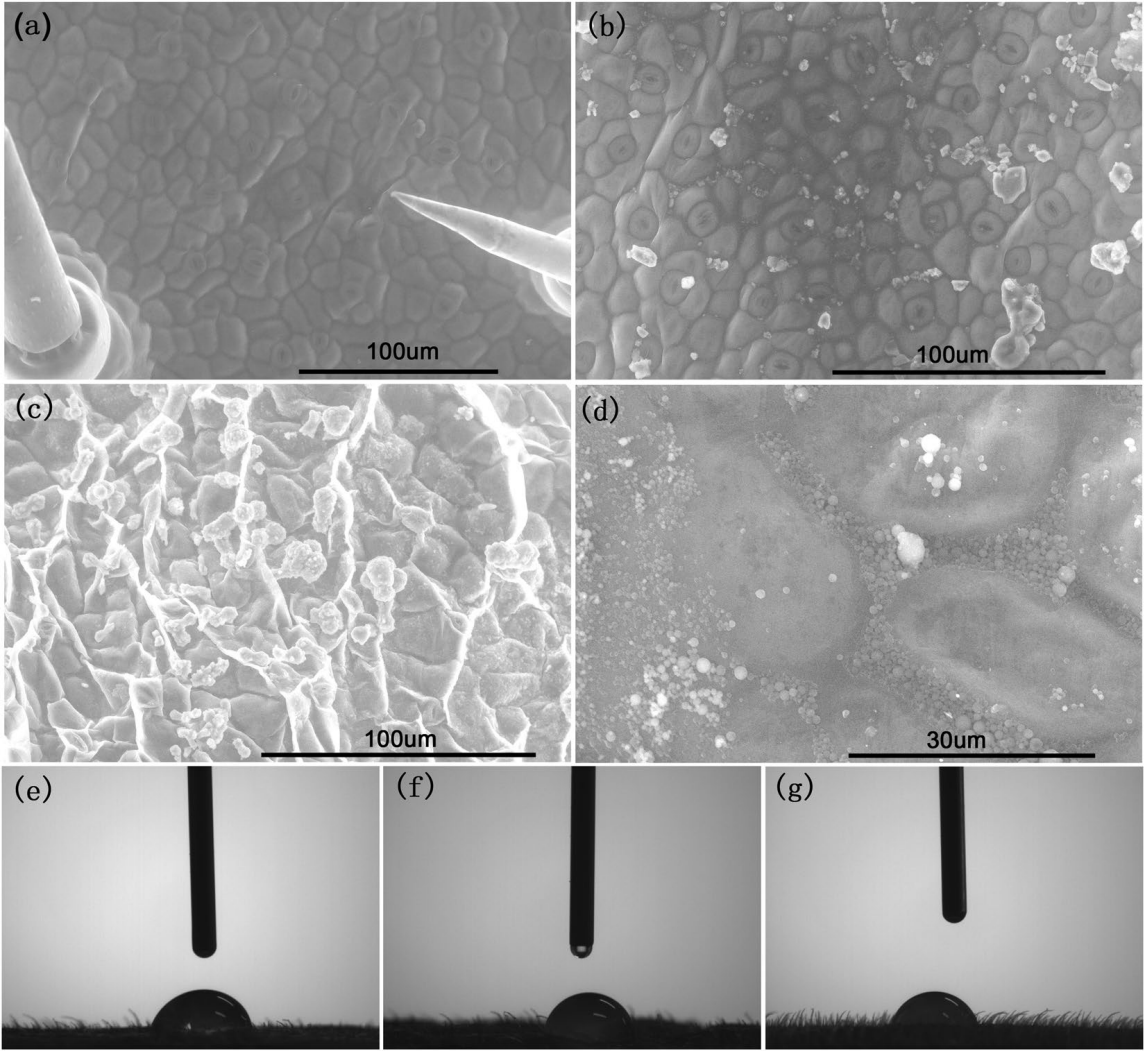
ESEM images (**a**–**d**) and contact angle (**e**–**g**) on cucumber leaf of the EMB slow-release microsphere and the commercial EMB formulations using a clean cucumber leaf as a control﻿ (**a**)﻿: the﻿﻿ commercial EMB WDG (**b**,**e**), the commercial microcapsule (**c**,**f**) and the EMB slow-release microsphere (**d**,**g**).

We investigated the dispersion and contact behaviour of microsphere droplets on cucumber leaves before and after drying by e﻿nvironment scanning electron microscopy (ESEM) and contact angle measurements (Fig. 8a–g), respectively. Microspheres were sprayed on cucumber leaves using the commercial EMB microcapsule and WDG as controls. ESEM images (Fig. 8a) clearly show the vein distribution on the surface of a clean cucumber leaf. As shown in Fig. 8, compared to the EMB WDG (Fig. 8b) and the commercial EMB microcapsule (Fig. 8c), the EMB microsphere (Fig. 8d) has a good dispersion property on the cucumber leaves. The dried active ingredient in the EMB slow-release microsphere was embedded between the veins due to their small size (Fig. 8d), which is conducive to reducing the loss and droplet drift of pesticides, and improving the utilization rate. In addition, the contact angles of the EMB microsphere droplets were also analysed as an evaluation criterion in the wettability research. As shown in Fig. 8e–g, the contact angles of the commercial WDG, commercial EMB microscapsule, and slow-release microsphere were 76.51°, 77.01°, and 76.01° on the cucumber leaves, respectively. The results illustrated that the contact angles of the EMB microsphere droplets were not significantly reduced compared with the commercial formulation, which may be due to the presence of a large amount of pesticide adjuvant in the commercial formulation. Although there was a very small amount of pesticide adjuvant in our microspheres, the similar contact angle on the cucumber leaves was retained due to the small size of the microsphere.

In summary, we prepared a controlled slow release microsphere for the widely-used insecticide EMB based on emulsion polymerization in order to improve its stability and efficacy. The influence of different surfactants on average size and on PDI of the EMB slow-release microsphere was investigated, and an optimal system was obtained using EL-40 as the surfactant. The optimal EMB microsphere has good slow-release performance and a longer activity period compared to the technical EMB and commercial WDG. Meanwhile, the EMB slow-release microsphere had significant anti-photolysis capability and good stability due to the protection afforded by the carrier encapsulation. Its small particle size and good dispersity also strengthen its deposition and distribution performance as a cucumber foliar spray, thus improving pesticide efficacy. Such advantages are conducive to reducing the number of times EMB needs to be sprayed and the effective dosage of EMB, potentially resulting in reduced environmental impacts.

## Materials and Methods

### Chemicals

Technical emamectin benzoate (technical grade active ingredient) was purchased from Hebei Veyong Bio-Chemical Co., Ltd., China. Poly(vinyl alcohol) (PVA) with a Mw of 30,000–70,000 and a hydrolysis of 87–89% was purchased from Sigma-Aldrich Shanghai Trading Co., Ltd. Polylactide (PLA) was purchased from NatureWorks. Calcium dodecylbenzenesulfonate (emulsifier 500), styryl phenol polyoxyethylene ether (emulsifier 600), alkylphenol formaldehyde resin polyoxyethylene ether (emulsifier 700), and phenylethyl phenol polyoxyethylene polyoxypropylene ether (emulsifier 1601) were purchased from Cangzhou Hongyuan Agrochemical Co., Ltd. Polyoxyethylene sorbitan monooleate (Tween 80) and polyethylene glycol sorbitan monostearate (Tween 60) were purchased from J&K Scientific Ltd. Sorbitan monooctadecanoate (span 60) and sorbitan (Z)-mono-9-octadecenoate (span 80), polyoxyethylene castor oil (EL-40), and aliphatic alcohol polyethylene oxide ether (AEO-9) were purchased from Haike Hong Chong Biotechnology Co. Ltd. (Beijing, China). The water used in all analytical experiments was Milli-Q water (18.2MΩ.cm, TOC ≤ 4 ppb) prepared with a Milli-Q Advantage A10 system (Millipore, Milford, MA, USA). Other chemical reagents were of analytical grade and were purchased from Haike Hong Chong Biotechnology Co. Ltd. (Beijing, China).

### Preparation of EMB slow-release microspheres

EMB slow-release microspheres were prepared using an oil-in-water (O/W) emulsion method combined with an ultrasonic and shearing physical emulsification process. Briefly, PLA and EMB were dissolved in methylene chloride as the oil phase. For the water phase, we choose ten different surfactants including EL-40, span 60, Tween 80, span 80, Tween 60, emulsifier 500, emulsifier 600, emulsifier 700, emulsifier 1601, or AEO-9, and mixed them with PVA aqueous solution. Then, the oil phase was dripped slowly into a large volume of the water phase under high shear emulsification (FA25, FLUKO, Ruhr-gebiet, Germany) to prepare a coarse emulsion. The coarse emulsion was further uniformly dispersed by ultrasonic emulsification (JY 92-IIN, SCIENTZ, Ningbo, China). After the uniform emulsion was obtained, it was solidified under magnetic stirring overnight (RW20, IKA, Staufen, Germany). The hardened EMB slow-release microspheres were collected via centrifugation and washed three times with deionized water. Centrifugal products were freeze-dried (FD-81, EYELA, Tokyo, Japan) to yield a free-flowing powder.

### Characterization of the EMB slow-release microspheres

Morphology of the EMB slow-release microspheres was investigated by scanning electron microscopy (SEM, JSM-6700 F, JEOL Ltd., Akishima-shi, Japan) with an accelerating voltage of 5 kV. Prepared samples were added dropwise onto the silicon slice surface and dried at room temperature, and then coated with a thin layer of platinum using a sputtercoater (EM ACE600, Leica, Vienna, Austria) for SEM measurement. The samples were also characterized by transmission electron microscope (TEM) (Philips 6 CM120, Netherlands). The sizes of EMB slow-release microspheres were measured at 25 °C with laser scatter using a zetasizer (Zetasizer NanoZS90; Malvern, Worcestershire, UK).

### Fourier transform-infrared (FT-IR) spectroscopy

FT-IR spectra of the technical EMB, PLA, and the EMB slow-release microsphere (EMB-PLA) were determined using a Thermo Nicolet Avatar-330 FT-IR instrument. Spectra data were collected in the transmission mode by averaging 32 scans over a wavenumber range of 4000−400 cm^−1^.

### Determination of pesticide loading content and entrapment efficiency

The loading content and entrapment efficiency of EMB in the slow-release microspheres was measured by fully dissolving the microspheres in methylene chloride. The final solution was dried via reduced-pressure distillation to obtain dry precipitation. The EMB precipitate was dissolved in methanol and EMB concentrations were determined by UV–vis spectrophotometry (TU901, Shimadzu Corporation, Kyoto, Japan) at a wavelength of 245 nm.

$$\begin{array}{rcl}Loading\,content & = & \frac{{\rm{total}}\,{\rm{mass}}\,{\rm{of}}\,{\rm{EMB}}\,{\rm{loaded}}\,{\rm{in}}\,{\rm{specimens}}}{{\rm{total}}\,{\rm{mass}}\,{\rm{of}}\,{\rm{specimens}}}\times 100 \% \\ Entrapment\,efficiency & = & \frac{{\rm{total}}\,{\rm{mass}}\,{\rm{of}}\,{\rm{EMB}}\,{\rm{loaded}}\,{\rm{in}}\,{\rm{specimens}}}{total\,mass\,of\,EMB}\times 100 \% \end{array}$$

### *In vitro* release of the EMB slow-release microspheres

The EMB slow-release property was evaluated using a dynamic dialysis method. EMB slow-release microspheres were dispersed in 10 mL methanol/water mixture in the dialysis membrane. The dialysis membrane was sealed into a brown flask with 90 mL of the mixed solution of methanol and water as the release medium. The flask was incubated in an incubator shaker at 300 rpm at 25 °C. At various time intervals, 5 mL solution outside the dialysis membrane was withdrawn and was replaced with a fresh mixed solution. The concentration of EMB was measured using a UV–vis spectrophotometer to determine the kinetic profile of release. The technical EMB (TC) and commercial WDG were used as controls.

### Stability studies

The UV-shielding properties of the EMB slow-release microspheres were tested with the technical EMB as a control. In details, the microspheres were dispersed and divided equally into culture dishes, which were illuminated under a UV lamp (500 W) with a maximum emitting light of 365 nm wavelength at 25 °C. At specified time intervals (12, 24, 36, 48, 60, and 72 h), the culture dish was taken out of the reactor and the EMB concentration of samples was analysed. A physicochemical stability test of the EMB slow-release microspheres was also conducted according to CIPAC MT 46 and GB/T 19136–2003. The samples were respectively packed in glass tubes and stored at 0 ± 2 °C for 7 days and 54 ± 2 °C for 14 days, and the changes in EMB loading contents in the slow-release microspheres were studied.

### Dispersion and contact property measurement on cucumber leaf

Cucumbers were cultivated in artificial climatic cabinets and were used as model plants to study the distribution and contact angle of the EMB slow-release microspheres. The commercial EMB microcapsule and WDG were used as controls. The samples were sprayed onto the surfaces of clean, live cucumber leaves and dried in air for environment scanning electron microscopy (ESEM; Quanta FEG 250; American) measurement to investigate the distribution of the microspheres on cucumber leaves. Contact angles of the EMB microsphere droplets on cucumber leaves were also measured with a contact angle apparatus (JC2000D2M; Zhongchen Digital Technology Apparatus; China). A piece of cucumber leaf was attached to the slide and a 5 μL droplet of each sample was deposited on the leaf by a microliter syringe and the contact angle image was captured immediately.

## Electronic supplementary material

Synthesis and characterization of emamectin-benzoate slow-release microspheres with different surfactants
