# Supplementary material for: Synthesis and characterization of emamectin-benzoate slow-release microspheres with different surfactants
Source: Sci Rep. 2017 Oct 6;7:12761. doi: 10.1038/s41598-017-12724-6 (PMC5630577; doi:10.1038/s41598-017-12724-6)
Supplement: Supplementary file 1 — Synthesis and characterization of emamectin-benzoate slow-release microspheres with different surfactants [file 41598_2017_12724_MOESM1_ESM.doc]

**Supplementary Information**

**Synthesis and characterization of emamectin-benzoate slow-release microspheres with different surfactants**

Yan Wang,^1,2^ Anqi Wang,^1,2^ Chunxin Wang,^1,2^ Bo Cui,^1,2^ Changjiao Sun,^1,2^ Xiang Zhao,^1, 2^ Zhanghua Zeng, ^1,2^ Yue Shen, ^1,2^ Fei Gao, ^1,2^ Guoqiang Liu, ^1,2^ and Haixin Cui^1,2,*^

^1^Institute of Environment and Sustainable Development in Agriculture, Chinese Academic of Agriculture Sciences, Beijing 100081, China.

^2^Nano Agricultural Research Center, Chinese Academic of Agriculture Sciences, Beijing 100081, China.

Corresponding Author

* (H.C.) Phone: 86-010- 82106013. E-mail: [cuihaixin@caas.cn.](../../../../F:%5C实验F盘%5C实验%20(F)%5C王琰-农科院%5C学生文章%5C学生文章%5C刘宝霞%5C文章2%5C最后投稿%5Ccuihaixin@caas.cn)

**This document file includes:**

**SUPPLEMENTARY FIGURES 1-2**

**SUPPLEMENTARY FIGURES**


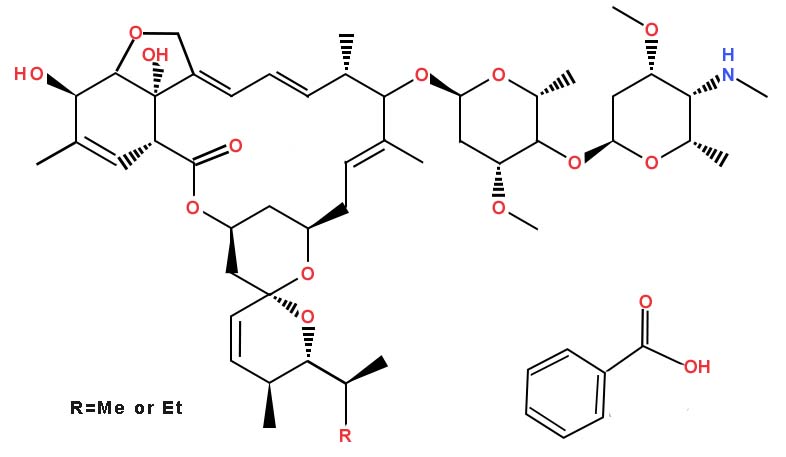


**Supplementary Figure 1.** **Chemical structure of emamectin benzoate**

**
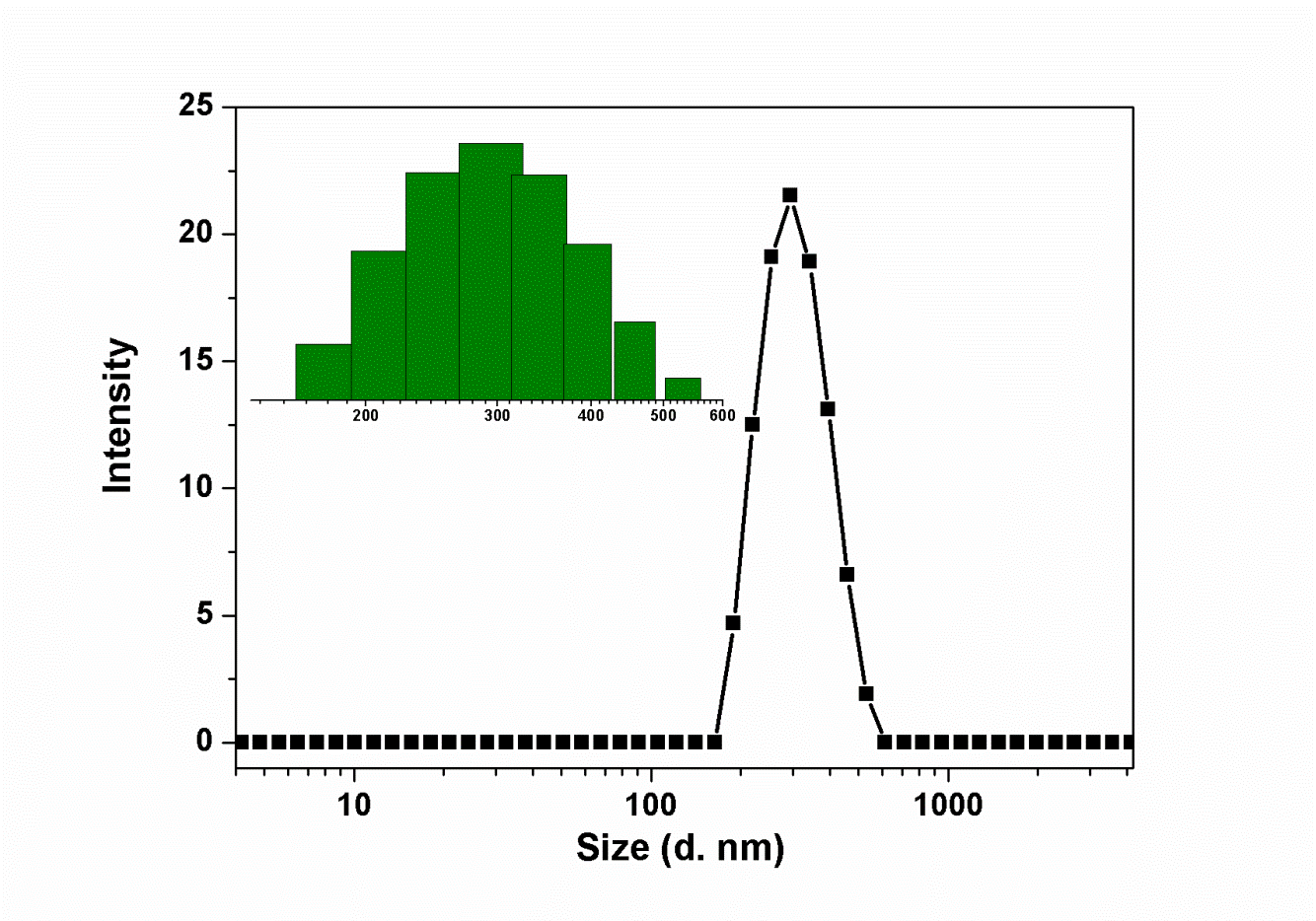
**

**Supplementary Figure 2.** **Particle size distribution of the optimal EMB slow-release microsphere measured by laser scatter.**
